# Dynamic Behavior of Corrugated Cardboard Edge Damaged by Vibration Input Environments

**DOI:** 10.3390/ma18184364

**Published:** 2025-09-18

**Authors:** Seungjoon Kim, Yeonjin Jang, Wanseung Kim, Changjin Lee, Junhong Park

**Affiliations:** 1 Department of Mechanical Engineering, Hanyang University, 222 Wangsimni-ro, Seongdong-gu, Seoul 04763, Republic of Korea; princel9@hanmail.net (S.K.); jyj960730@gmail.com (Y.J.); xqxwxexr@naver.com (W.K.); 2 LG PRI, 222 LG-ro, Jinwi-myeon, Pyeongtaek-si 17709, Gyeonggi-do, Republic of Korea; changjin80.lee@lge.com

**Keywords:** dynamic Stiffness, vibration damage, Basquin’s power law, corrugated cardboard, expanded polystyrene (EPS), packaging

## Abstract

This study investigates the dynamic performance and degradation behavior of corrugated cardboard used as protective packaging for home appliances subjected to random vibrations during transportation. Simulated vibration tests were conducted on fully packaged refrigerators to assess the mechanical response of cardboard and expanded polystyrene (EPS) supports under prolonged vibration excitation. Relaxation tests were performed to characterize time-dependent stress decay in the absence of vibration, while cantilever beam experiments quantified dynamic stiffness degradation during vibration exposure. The vibration-induced damage was evaluated by monitoring the decrease in support stiffness over time, revealing a distinct exponential reduction that correlated with increasing excitation levels. Statistical load count analyses, based on auto-spectral methods and Basquin’s power law, were used to model fatigue behavior and predict service life. The findings demonstrated that corrugated cardboard exhibited comparable performance to EPS in maintaining support stiffness while offering the advantage of environmental sustainability. These results provide quantitative evidence supporting the use of cardboard as an effective and eco-friendly alternative to polymer-based packaging materials, contributing to the development of optimized packaging solutions with enhanced vibration durability.

## 1. Introduction

The proper packaging of constituting components is essential for ensuring the safe transport of commercial products, as it efficiently absorbs mechanical shocks without causing permanent defects. Conventional packaging relied on polymer materials of expanded polystyrene (EPS) and Styrofoam. The use of corrugated cardboard is rapidly increasing due to its eco-friendly nature [1,2], along with its cost-effectiveness and superior performance in isolating external mechanical impacts. This is especially relevant in the transportation of home appliances, where shelves are supported and secured by flexible pads. Shelf-ready packaging (SRP) not only reduces installation time after delivery from the factory to the customer but also helps minimize errors during setup, preventing unnecessary returns or service calls for these appliances. For SRP to be effective, shelves must remain securely fixed during transport and be easily removable upon delivery. During transit of packaged home appliances from manufacturing sites to their destinations, they are subjected to various vibrations caused by transportation vehicles such as trucks, ships, and airplanes. These vibrations, particularly random vibrations from engines or road irregularities, can persist throughout the delivery process. If the shelves become dislodged due to vibration exposure, this can result in product damage, leading to unnecessary returns and deterioration of product quality. Therefore, packaging structures must be designed to securely hold shelves in place during transit, while also allowing easy removal upon delivery. Corrugated cardboard has become widely used for this purpose due to its favorable characteristics, with its applications expanding across industries. The anisotropic properties of corrugated cardboard, determined by the shape and orientation of its internal flutes, necessitate robust testing and evaluation methods to assess potential fatigue failures caused by vibrations. Ensuring vibration durability under these realistic conditions underscores the industrial and environmental importance of this study.

Evaluation of the elastic behavior of corrugated cardboard using experimental methods combined with analytical modeling was proposed to understand the anisotropic stiffness responses due to its layered geometry [3]. A large strain elastoplastic constitutive model was used to accurately simulate the nonlinear behavior of corrugated board under complex loading, extending its applicability beyond simple elastic assumptions [4]. A nonlinear elastic plate theory tailored to thin fibrous materials was developed, providing a theoretical analysis for characterizing paper-based structures [5].

Advanced modeling techniques such as homogenization have been employed to simplify the complex geometry of corrugated boards into equivalent orthotropic layers. Homogenization for double-wall corrugated boards under bending and shear, achieving accurate yet computationally efficient models was proposed [6]. Suarez et al. [7] extended this approach to simulate seating furniture using multi-wall corrugated cardboard. Numerical predictions of structural properties are needed for applications in relevant fields of interest [8,9,10]. The properties including bending stiffness, edge crush resistance, and compressive strength estimates should be considered. The buckling and collapse of corrugated board under multi-axial stress were addressed by Patel et al. [11] and Garbowski et al. [8]. The failure of mechanisms for edge-loaded designs was considered. Transverse shear effects in orthotropic plate buckling analysis, relevant for thicker or multiply boards were analyzed to understand the failure [12]. The influence of crushing during manufacturing or handling on the overall strength of fiberboard, providing a link between damage accumulation and functional performance was investigated [13].

Experimental studies form the cornerstone of model validation and performance benchmarking. Detailed experimental studies and finite element simulations on corrugated packaging for fresh produce, evaluating mechanical response under various environmental conditions such as humidity and temperature were performed [12,13]. Micro-wave configurations in board design were studied using both experiments and Finite element method (FEM) to determine how wave geometry affects stiffness and crushing strength [14]. Corrugated box design principles for horticultural products, combining structural, economic, and environmental aspects were investigated [15]. Non-local sensitivity analysis and numerical homogenization for optimal packaging design considering the influence of geometry and material distribution were performed [16]. The effects of perforation patterns on compressive strength while maintaining structural integrity were analyzed [6]. Most of these studies have emphasized static or short-duration loading, environmental influences, or structural optimization. Systematic experimental evaluation of long-term stiffness degradation under random vibration representative of transport environments remains limited; this industrial necessity motivated the present study.

Life cycle assessments comparing corrugated cardboard with alternative materials such as polystyrene and bioplastics were performed [17,18]. The cardboard-based systems were favored in terms of environmental impact, especially when combined with recycled content and bio-coatings. Beyond packaging, the structural efficiency of corrugated cardboard as a building material was investigated [19]. Physical testing protocols in paper and cardboard are available as handbooks [20]. To use corrugated cardboard as packaging material, the dynamic properties including bending and longitudinal stiffness, vibration reduction, and structural integrity under various environmental conditions should be understood. The packaging for transport parts should protect the products from external vibratory disturbances during delivery. The vibration damage induces minute progressive failure of the packaging material and reduces the protective performance of packaged goods. Corrugated cardboard, owing to its lightweight, recyclability, and structural efficiency, has become a prevalent material in packaging of home appliances. Understanding its mechanical behavior and structural re-liability under vibratory environmental conditions is crucial for optimizing performance and secured reliability. In addition to the mechanical testing, the proper evaluation method to investigate the vibration damage of the packaging material is required for optimizing the constituting components. Without such information, vibration test experiments to the packaged product should be performed for structural integrity inspections of packaged products. These considerations were incorporated in this study with laboratory experimental assessment specially designed for the vibration integrity inspection.

This study investigates the effectiveness of corrugated cardboard packaging in protecting products from transport-induced random vibration. Simulated vibration tests were conducted on fully packaged refrigerators, and the vibration response was measured to assess potential loosening or displacement of internal components. The experiments were performed to evaluate packaging performance degradation under edgewise loading and to measure stress relaxation behavior after compression. The corrugated cardboard packaging exhibited minor vibration-induced deformation in the fluted structure without catastrophic buckling. The vibration response of the internal shelf differed between packaging types, indicating that the supporting method influenced vibrational energy transmission; the cardboard dissipated some input through structural flexing. Repeated vibrations may compromise packaging integrity. These findings highlight the strengths and limitations of corrugated cardboard packaging and inform design improvements—via material combinations or design modifications to enhance protection against vibration-induced damage and ensure safe transport of commercial products. Given the growing focus on eco-friendly materials, packaging design is likely to evolve toward more efficient and sustainable solutions.

## 2. Vibration Induced Damage on the Cardboard During Refrigerator Transportation

**Figure 1:**
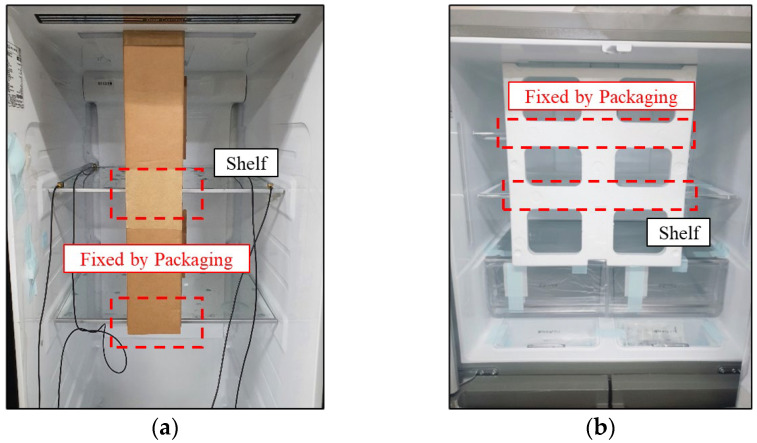
Shelf-ready packaging for refrigerator’s inside.: (**a**) corrugated cardboard; (**b**) eps (expanded polystyrene).

Home appliances, such as refrigerators, are exposed to various vibrations during transportation. To simulate vibration-induced fatigue failures that occur during delivery, a range of methods is employed [21]. Once production is completed, the product is transported to the destination via trucks, ships, and airplanes before reaching the consumer. After delivery is completed, no defects should occur during subsequent processes such as installation. Therefore, it is essential to implement design measures that prevent the possibility of defected parts, and it must be possible to assess defect occurrence under laboratory conditions. To monitor defects arising during transportation in a controlled laboratory environment, accelerated durability tests are conducted on packaged products using large-scale vibration exciters. In the case of refrigerators, supporting structures are required to minimize damage and defects caused by vibrations during transportation. As a commonly used approach, protective stabilizer packaging is shown in Figure 1 is applied as a temporary support structure to secure fragile or movable components of a product during shipping and transportation. It prevents damage caused by vibrations, impacts, or shifts that can occur during transportation to customers. The stabilizers are commonly made of plastic brackets, foam padding, paper cardboard (Figure 1a), EPS (Figure 1b), or adhesive tapes. It prevents internal parts including shelves, drawers, or glass panels from moving or breaking. These comments are re-moved and discarded during installation. Such supporting structures, when applied on the refrigerator, must be capable of evaluating vibration durability to minimize damage that may occur during transportation.

**Figure 2:**
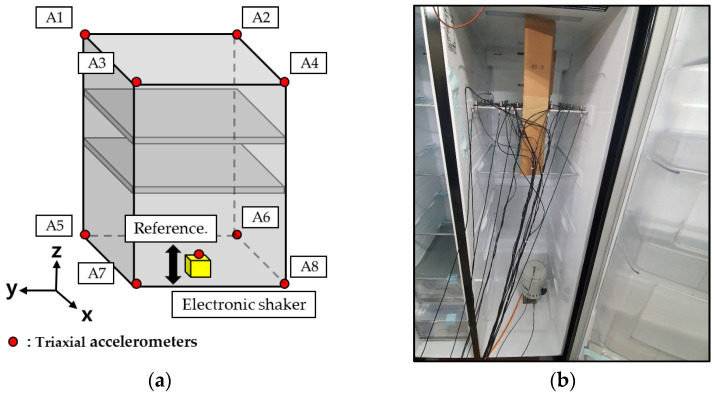
Experimental setup for vibration tests of assembled refrigerator: (**a**) schematic of accelerometer and shaker attachment inside a refrigerator; (**b**) refrigerator used for the experimentations.

To investigate the vibration characteristics of refrigerators when exposed to mechanical excitation, vibration durability tests were conducted in a laboratory environment. Figure 2 shows a photograph of the refrigerator used in the experiment. Based on common vibration spectra observed in transport environments, an inertia motor shaker was installed on the floor to apply vibration inputs simulating those occurring during shipment. To measure the vibration transmitted to the shelf during excitation, tri-axial accelerometers were attached to the shelf. Random vibrations were applied, with the objectives of identifying the dominant frequency ranges responsible for vibration-induced damage and clarifying the mechanisms by which such damage occur.

In particular, the focus was on observing how cumulative external damage arising from prolonged vibration exposure affects the vibration characteristics. To examine the influence of protective stabilizers, experiments were carried out with two types of support structures, as illustrated in Figure 2: one constructed from corrugated cardboard and the other using EPS. EPS is commonly employed as support material; however, continuing efforts are underway to replace it with environmentally friendly paper cardboard structures.

**Figure 3:**
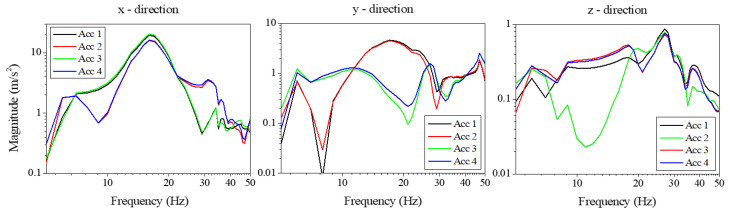
Vibration response of shelf supported by paper package. The resonant vibration of the shelf appeared at the corresponding resonance frequency of the refrigerator. The vibration resonance of the shelf itself, especially in the vertical directions appeared at frequencies larger than 40 Hz.

To monitor the motion of the shelf when the refrigerator was subjected to vibrations during transportation, tri-axial accelerometers were affixed to the shelf, and the resulting vibrations were compared with those of the refrigerator’s frame itself. Vibration measurements obtained by attaching accelerometers to the main frame revealed that when the refrigerator was supported on the floor, natural modes of the structural frame occurred at 7 Hz, 17 Hz, and 27 Hz. These natural vibration modes were determined by the rigidly designed frame and were not significantly affected by external excitation. Shear deformation associated with these frame modes was transmitted through the support points to the shelf vibrations. Figure 3 shows the measured vibration responses at the four corners where the shelf is connected to the refrigerator. The input vibration amplitude at each shelf support varied, reflecting the influence of the refrigerator mode shapes, and excitation was observed along all three axes. Dominant vibration resonances appear at 7 Hz and 17 Hz in the *x*–*y* directions, and at 7 Hz, 17 Hz, and 27 Hz in the *z* direction. These observations indicate that the 7 Hz and 17 Hz vibration modes correspond primarily to lateral movements of the frame, whereas the 27 Hz mode is consistent with a torsional displacement. In particular, the amplitude and phase differences among the four shelf-corner sensors at 27 Hz support this observation.

**Figure 4:**
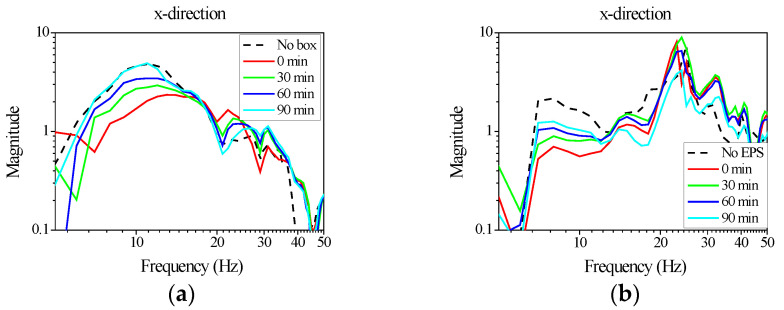
Effects of vibration induced damage on the vibration responses measured on the refrigerator shelf fixed with different packaging structures: (**a**) paper-corrugated board (box) and (**b**) EPS.

Unlike the refrigerator frame modes, the natural vibration modes of the shelf itself occurred above 34 Hz, in which the vibration amplitude was highest at the center of the shelf panel and lowest along the z-axis near the corners. During prolonged exposure to external vibration inputs, the properties of the stabilizers attached for fixation changed, resulting in variations in shelf vibration amplitudes due to altered support conditions. Sustained vibration over time led to progressive changes in the stabilizer’s material properties, particularly for those fabricated from paper or EPS, which were more susceptible to property degradation than plastic structural supports. Figure 4 presents the observed vibration responses of the shelf subjected to shaker-induced excitation in the laboratory. Random vibration in the frequency range of 5–200 Hz was applied during testing. Figure 4a shows the results for the cardboard–supported configuration, while Figure 4b depicts the EPS-supported case. In both scenarios, changes in shelf vibration were observed as excitation continued. Notably, at 7 Hz, the amplitude of shelf vibration increased with exposure duration, whereas vibration amplitudes in frequency ranges above 20 Hz decreased over time. When considering failure scenarios involving detachment as vibration accumulated, this decreasing trend in higher-frequency vibrations contrasted with expectations based on detachment mechanisms. From these measurements, it was inferred that the vibration responsible for detachment originated from the low-frequency deformation of the refrigerator occurring around 7 Hz. As the exposure time increased, the band-limited response near 7 Hz grew systematically, indicating a progressive reduction in the support stiffness of the stabilizers; consequently, the likelihood of shelf disengagement under sustained vibration increased. This trend is consistent with the vibration mode observation shown in Figure 3, where low-frequency frame-dominated motion governs the energy transmitted to the shelf.

**Figure 5:**
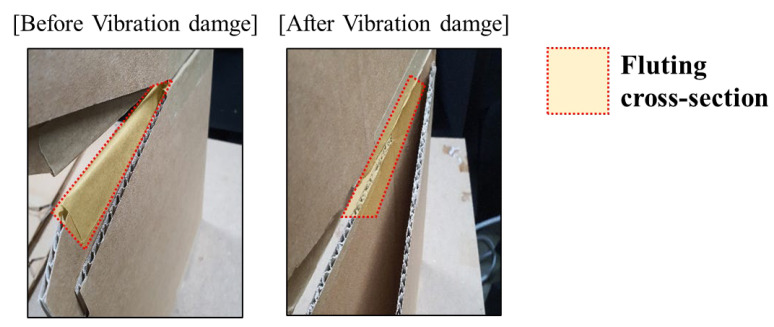
Vibration induced damage on the fluting cross-section. The vibration induced distortion in the surface without buckling of the board.

Figure 5 shows cross-sectional views of the cardboard before and after the vibration tests. As testing progressed, visible wear and compression of the cross-sections were observed. Visual inspections were also carried out, and any cracking, permanent deformation, or delamination in the samples after vibration exposure was observed. As the cardboard was crushed, its effective length decreased in the edgewise direction. The incremental reduction in edgewise length weakened the stiffness supporting the shelf, resulting in increased low-frequency vibration amplitudes that promoted detachment phenomena. Although wear-induced frictional damping increased at frequencies above 20 Hz, leading to reductions in shelf vibration amplitudes in these ranges, this effect was minimal with respect to the occurrence of detachment. This indicated that high-frequency vibrations did not significantly affect the displacement response of the shelf. The wear-related vibration-induced degradation in material properties was similarly observed for EPS stabilizers. To ensure sufficient vibration durability of stabilizers, design approaches that minimize such wear effects are necessary. Accordingly, a vibration durability test setup was developed to evaluate the performance of stabilizer materials under simulated transport conditions.

## 3. Experimental Methods for Vibration Damage Evaluation of Packaging Materials

### 3.1. Relaxation Tests to Measure Decay of Support Stress After Installation

In vibration durability testing, it is necessary to measure the time-dependent changes in the material properties. As an initial step, a relaxation test was conducted as a material characterization method to observe how the supporting force changes over time in the absence of external vibration. The packaging materials used in the refrigerator vibration tests were tested. The cardboard showed a flute height of 5 mm, consisting of two fluted medium layers and paperboard facings, sized to 150 mm × 150 mm. The EPS foam samples were cut to the same plan dimensions and a comparable thickness (30 mm) to provide a fair comparison of cushioning thickness.

**Figure 6:**
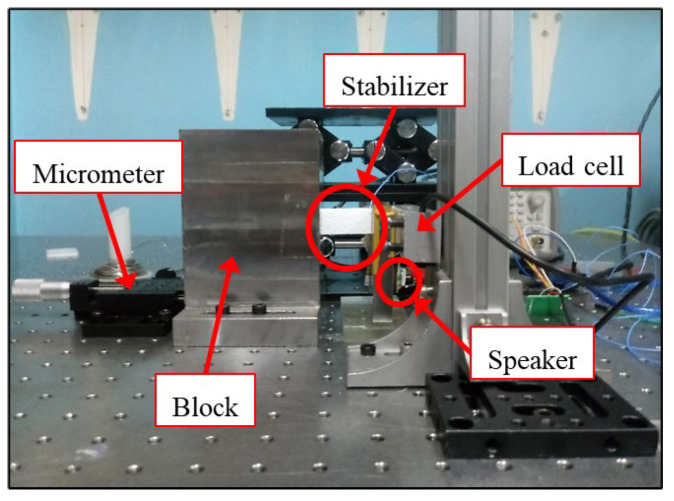
Laboratory setup for measuring stress relaxation of the stabilizer material under edge compression.

The relaxation tests with the setup shown in Figure 6 were performed to establish a baseline before vibration testing. Samples were compressed to a fixed deformation and held, while the decay in stress was recorded over time. The corrugated pad specimens were loaded in the edgewise direction (flutes vertical) to 2% strain of their original thickness, a strain level within the elastic range but significant enough to engage the material’s internal structure. EPS specimens were similarly compressed to 2% strain. Once the target deformation was reached, the crosshead position was held constant. The force on the specimen was logged at 1 Hz for a period of 10 min. This test reveals how quickly each material relaxes (loses stress) under constant strain, which is a measure of its viscoelastic and time-dependent deformation characteristics.

**Figure 7:**
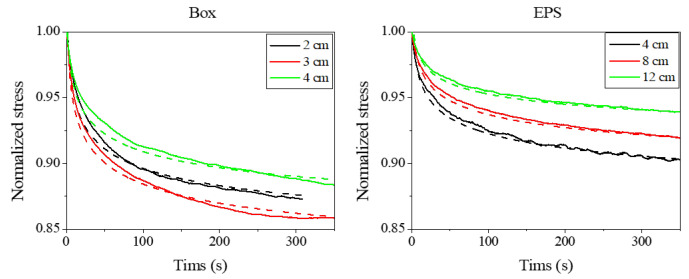
Variation in stress with time after compression with compression ratio of 2%. Solid Line: measured stress variation, dashed line: its fitted curve using an exponentially decreasing stress variation.

Figure 7 presents the measured stress of the specimens measured for different specimen thicknesses. The dashed line indicates the fitted curve representing an exponentially decreasing stress variation. This result demonstrates that stress re-laxation occurred, consistent with the behavior typically observed in polymeric materials. The tendency for relaxation was similar between the paper cardboard and the EPS materials. The final converged stress values were approximately 85% for the paper cardboard and 95% for the EPS. The stress did not decrease below these values, indicating that sufficient residual force remained to support the shelf.

While performing the same relaxation test, additional experimentation was conducted by applying external vibration to the compression plate as shown in Figure 6 to observe whether the form of relaxation changed due to vibratory excitation. Significant differences were not observed under additional vibration, indicating that relaxation tests alone cannot distinguish vibration-induced damage. These results provide useful baseline information on the time-dependent stress relaxation of the materials and highlight the need for separate vibration durability evaluation through dynamic stiffness monitoring. Consequently, an alternative approach was adopted: monitoring changes in the vibration stiffness of the support structures during actual tests, where variations in supporting stiffness attributable to vibration damage had already been observed. This approach allowed the assessment of how vibration damage influences the dynamic stiffness of the support structures.

### 3.2. Vibration Test Method to Determine Cardboard Support Stiffness Properties

**Figure 8:**
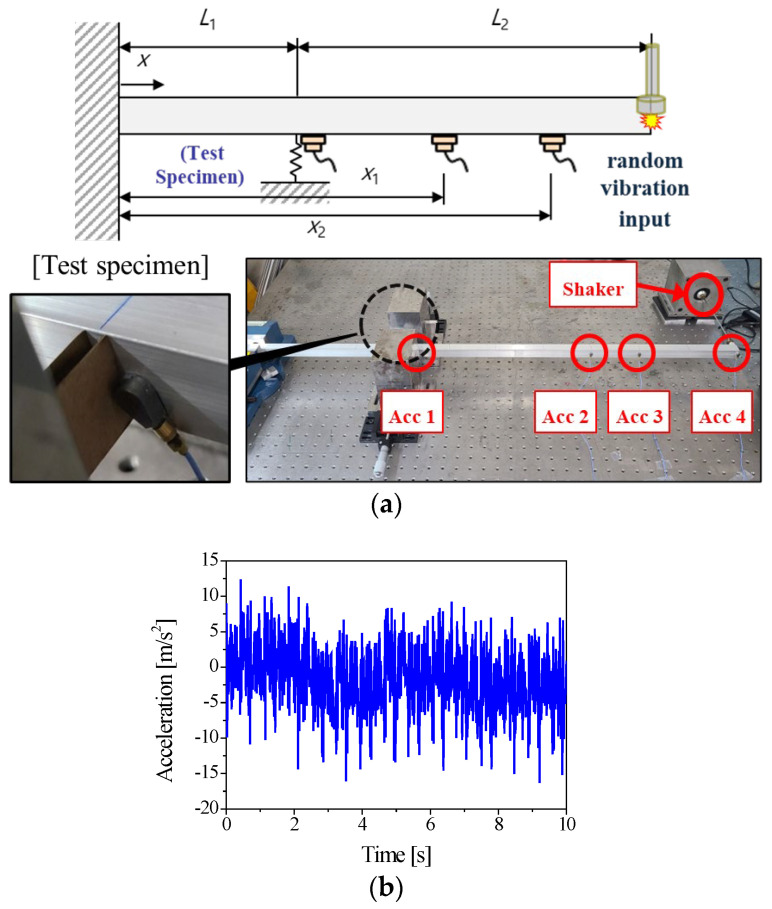
Experimental setup to measure vibration induced damage on the package in edge direction. (**a**) Schematic and picture of the experimental setup; (**b**) excitation force time history.

Figure 8 shows the experimental setup used to measure the dynamic stiffness of the cardboard in the edge direction under laboratory conditions. The room temperature was maintained at 19–23 °C and the relative humidity at 50%. In this configuration, the cardboard specimen supports the midsection of a cantilever beam. The vibration characteristics of the cantilever beam vary depending on the stiffness of the cardboard. By measuring the vibration response, the dynamic properties of the cardboard are directly monitored with progressive accumulation of the vibration damage. This experimental setup enables the quantitative characterization of the support stiffness of cardboard when it is employed as a packaging material. It is required to measure the stiffness variation in the cardboard with time after compression of the specimen. The Euler-Bernoulli beam based on linear elasticity theory considers only displacement in the direction of bending by neglecting shear deformation and rotation. The characteristics of the beam result in linear behavior. For bending direction vibrations, the governing equation is defined as follows:

(1) $$\frac{\hat{E}I\partial^{4}\hat{w}}{\partial \xi^{4}}+\rho A\partial^{2}\hat{w}/\partial \xi^{2}=F$$

where *w* is a transverse displacement, *E* is complex young’s modulus, *I* is the moment of inertia, *ρ* is a density, and *A* is a cross-sectional area. When the beam is supported by a stiffness element, the transverse displacement can be represented as:

(2) $$\begin{array}{cc} \hat{w}\left(\xi \right) & =\left[{\hat{A}}_{1}{sin\hat{k}}_{b}\xi +{\hat{A}}_{2}{cos\hat{k}}_{b}\xi +{\hat{A}}_{3}e^{{\hat{k}}_{b}\left(\xi -L_{2}\right)}+{\hat{A}}_{4}e^{{\hat{k}}_{b}\xi }\right]H\left(L_{1}-\xi \right)\\ & +\left[{\hat{A}}_{5}{sin\hat{k}}_{b}\left(\xi -L_{1}\right)+{\hat{A}}_{6}{cos\hat{k}}_{b}\left(\xi -L_{1}\right)+{\hat{A}}_{7}e^{{\hat{k}}_{b}\left(\xi -L_{1}-L_{2}\right)}+{\hat{A}}_{8}e^{{\hat{k}}_{b}\left(\xi -L_{1}\right)}\right]\\ & H\left(\xi -L_{1}\right) \end{array}$$

here *H* is a Heaviside step function, $L_{1}$ is the length of the specimen from the fixed end, ${\hat{k}}_{b}={\left(\rho A{\overset{̑}{\omega }}^{2}/\hat{E}I\right)}^{1/4}$ is the wavenumber of a beam. The coefficients ${\hat{A}}_{1}\sim {\hat{A}}_{8}$ are calculated by applying the eight boundary conditions at the fixed end, free end, and the point of stiffness support. From the vibration response, the transfer function is obtained as follows:

(3) $$\Lambda_{1}e^{i\phi_{1}}=\hat{w}\left(\xi_{2}\right)/\hat{w}\left(\xi_{1}\right)$$

To investigate dynamic properties of the packaging structure, a vibration test setup was designed as shown in Figure 8a. An aluminum beam with dimensions of 800 mm in length, 50 mm in thickness, and 20 mm in width is fixed at one end. Vibration was applied to the other end using an electric shaker (Brüel & Kjær, Type 8202, Nærum, Denmark). In the experimental setup, the test sample was securely fixed at 200 mm away from the fixed end.

The force induced in the sample during vibration excitation was measured using a force sensor (PCB Piezotronics, Model 208C02, Depew, NY, USA). The changing vibration characteristics under sustained random vibration input were monitored with an accelerometer (Brüel & Kjær, Type 4517, Nærum, Denmark). Acceleration measurements were obtained at three locations positioned 400 mm, 600 mm, and 800 mm from the fixed end. To examine the dynamic properties corresponding to the level of vibration damage, the time-dependent changes in vibration response were recorded as a function of the exposure duration. The support stiffness of the package specimen significantly influences the vibration characteristics. This translational stiffness increases the natural frequency compared to those without packages. The stiffness was quantified from the frequency response of the cantilever beam structure after wave propagation analysis in Equation (2). In contrast to the relaxation tests, changes in the support characteristics induced by vibration input were successfully observed when the vibration tests were utilized. These results are presented in detail in the following section, together with the external disturbance load count data.

### 3.3. Random Vibration Damage Quantification Analysis

To evaluate the stiffness degradation induced by random vibration input, it was necessary to count the number of occurrences of externally applied load forces. For measuring the external forces generated by random vibrations, their statistical characteristics were quantified using the auto-spectrum. After applying these external forces over a defined period, the support stiffness was continuously monitored to assess the influence of vibration-induced damage. The root-mean square (RMS) of the random response represents the excitation level applied to the package structure as

(4) $$G_{rms}=\sqrt{\int_{0}^{\infty }G(f)df}$$

where *G* is the power spectral density (PSD) of random excitation input. For damage evaluation, it is required to count cycles in the given amplitude. The *n*th spectral moments were calculated [22] as follows:

(5) $$m_{n}=\int_{0}^{\infty }f^{n}\cdot S(f)df$$

where *S* is the compression load measured between the end plate and the stack. The spectral moment is used to estimate the expected number of upward zero crossings, *E*[0] and the expected number of peaks, *E*[*P*] as [23]:

(6) $$E[0]=\sqrt{m_{2}/m_{0}},$$

(7) $$E[P]=\sqrt{m_{4}/m_{2}}$$

The irregularity factor, *γ*, is defined as

(8) γ=E[0]/E[P]

The number of force cycles expected in time is calculated as follows:

(9) N(S)=E[P]⋅p(S)⋅T

where *T* is the vibration excitation duration and *p* is the probability density function of the force range derived as follows [24]:

(10) $$p(S)=\left(\frac{D_{1}}{Q}e^{\frac{-Z}{Q}}+\frac{D_{2}\cdot Z}{R^{2}}e^{\frac{-Z^{2}}{2\cdot R^{2}}}+D_{3}Ze^{\frac{-Z^{2}}{2}}\right)/\sqrt{{4m}_{0}},$$

where $D_{1}=2(X_{m}-\gamma^{2})/(1+\gamma^{2})$, $D_{2}=(1-\gamma -D_{1}+{D_{1}}^{2})/(1+R)$, $D_{3}=1-D_{1}-D_{2}$, $Z=S/\sqrt{{4m}_{0}}$, $Q=1.25(\gamma -D_{3}-D_{2}\cdot R)/D_{1}$, and $R=(\gamma -x_{m}-{D_{1}}^{2})/(1-\gamma -D_{1}+{D_{2}}^{2})$.

Dirlik’s method utilizes the weighted sum of the Rayleigh, Gaussian, and exponential probability distributions and allows load count calculations when spectrum levels are given [24]. This approach is appropriate for counting the magnitude of disturbances when random loading is applied over prolonged durations.

### 3.4. Fatigue Relation Using Basquin Power Law S-N Curve

When exposed to random vibrations, the effective length of the support structure decreases due to the permanent deformation of the stabilizer package. This reduction in supporting stiffness has become a primary cause of shelf detachment. To define the external forces responsible for damage, the stiffness degradation ratio was employed as a quantitative indicator. The Basquin power law was used to present a fatigue model for the reduction in the support stiffness of the stabilizer package due to vibration-induced damage [25]. The Basquin power law was used to quantify fatigue to analyze vibration damage [25,26]. The Basquin power law fatigue relation is defined as follows:

(11) $$\sigma_{a}=a(2N_{f}{)}^{b}$$

where $\sigma_{a}$ is the external force RMS amplitude of the failure occurrence, a is the fatigue strength coefficient, b is the fatigue strength exponent, and $N_{f}$ is the reversal coefficient to failure. The fatigue model coefficient of each stabilizer package was derived based on the number of cycles when the support stiffness was reduced by 5%. This criterion can be adjusted depending on the specific application. Furthermore, the results derived using this approach serve as a basis for evaluating the support characteristics and performance of the packaging.

## 4. Experimental Results of the Vibration Damage Evaluation of Packages

**Figure 9:**
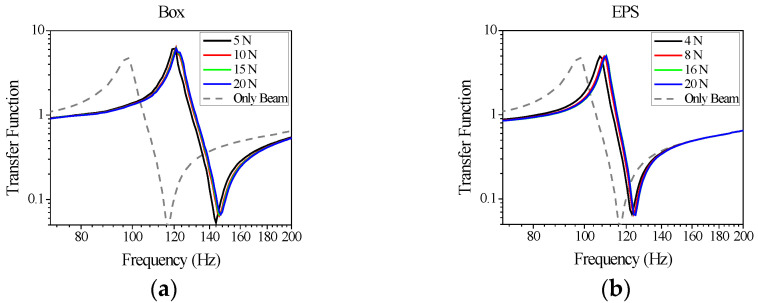
Measured variation in the vibration response of the supported beam by the package (**a**) Box and (**b**) EPS.

Through this study, the experimentally derived results quantifying vibration-induced permanent damage were validated. Figure 9 shows the vibration transfer function measured using the vibration test apparatus. To avoid dependency on varying excitation magnitudes during the experiments, frequency responses were derived from accelerometers positioned near the fixed end, where linear behavior was most evident. Figure 9 presents the vibration transfer functions of the cantilever beam structure supported by the packaging materials [27]. Specifically, Figure 9a shows the results for Box packaging, and Figure 9b shows those for EPS packaging. The packaging was pre-compressed with various static forces prior to vibration tests. Subsequently, vibration was applied to the cantilever beam, and the changes in vibration characteristics over time were monitored. Over time, a decrease in the natural frequency was observed, accompanied by an increase in damping. From these variations in dynamic behavior, the numerical methods used to derive these dynamic characteristics were employed [28].

**Figure 10:**
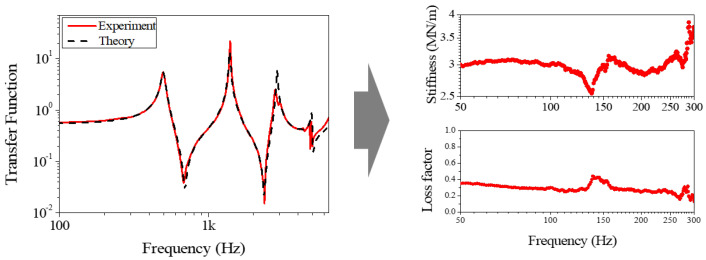
Measured dynamic stiffness and loss factor for EPS packaging, derived from the vibration transfer function.

The stiffness was measured as increasing consistently with frequency. EPS, as a homogeneous material, exhibited stiffness comparable to that of conventional closed foam materials. For the paper package, the stiffness in the edge di-rection was of particular importance and was observed to be higher than that of EPS. The loss factor, which represents the vibration-damping capability, was measured in the edge direction. This value was lower than that measured for EPS. Figure 10 presents the dynamic stiffness and loss factor derived from the measured vibration transfer function for the supported beam with EPS packaging. These plots demonstrate how these dynamic characteristics (stiffness and damping) are quantitatively obtained from the vibration response. Both stiffness and the loss factor varied over time. Notably, the reduction in stiffness was attributed to localized surface damage, which served as the primary cause of detachment. This behavior was not observed in the relaxation tests, demonstrating that the vibration testing method proposed in this study enables the evaluation of the durability performance of packaging structures under vibration exposure.

To track this behavior over time, the dynamic properties were averaged over frequencies below 400 Hz. The evolution of this average as a function of elapsed time since initial installation was recorded. Additionally, by varying the magnitude of the applied vibration, the influence of external excitation on stiffness degradation was systematically investigated. For each condition, a single long-duration run was conducted to capture time-dependent degradation; accordingly, run-to-run error bars are not reported. Input-PSD stability and sensor drift were continuously monitored during each run.

**Figure 11:**
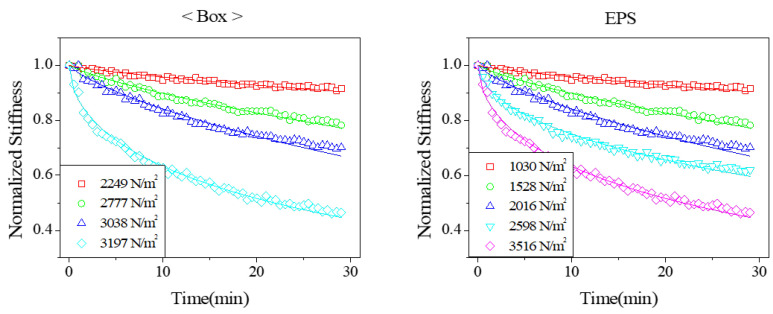
Measured variation in the dynamic stiffness due to vibration exposure with different stress magnitudes.

As vibration was applied, the supporting stiffness was observed to decrease over time. To quantify this reduction, the amount of degradation was calculated relative to the initial stiffness. Figure 11 illustrates the progression of stiffness reduction. The plot shows the proportion of stiffness loss due to vibration-induced damage, expressed as a ratio relative to the initial stiffness measured before vibration was applied. When the magnitude of external vibration was low, the reduction in stiffness followed a trend like that observed in the relaxation tests, decreasing gradually over time. The decreasing trend was exponential, and the solid line indicates the exponential function fitted to the measured data. A clear exponential decay was consistently observed.

When the magnitude of the external vibration input increased, the rate of stiffness reduction accelerated rapidly. This pronounced degradation was attributed to surface damage developing in the supporting structure. The reduction rate showed a strong correlation with the material’s loss factor. Notably, the reduction trends for the cardboard box and EPS supports were similar, demonstrating that cardboard can effectively replace EPS as a supporting material.

**Figure 12:**
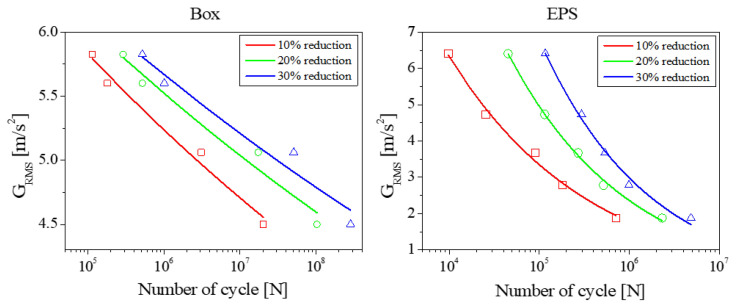
*N* curves constructed based on vibration-induced stiffness reduction in the packaging materials.

Figure 12 presents the $G_{rms}$-*N* curves for both EPS and cardboard box materials. As shown in these plots, the fatigue characteristics of EPS and cardboard box exhibited similar behaviors under vibration exposure. This observation confirms that stiffness, a critical mechanical property, can degrade significantly even without complete structural detachment, and the degree of stiffness degradation directly correlates with fatigue damage accumulation. This correlation enables the establishment of fatigue failure criteria based on stiffness reduction.

More specifically, the results indicate that setting higher stiffness degradation thresholds (e.g., 30% stiffness reduction) leads to fatigue failure after significantly more vibration cycles. Conversely, lower degradation thresholds (e.g., 10% stiffness reduction) resulted in earlier fatigue failure. This quantitative analysis aligns with the experimental results discussed previously, which demonstrated a clear exponential reduction in support stiffness that accelerates with increased vibration magnitude and exposure duration. The observed similarity in degradation rates between cardboard and EPS support further confirms that corrugated cardboard can serve as a viable, environmentally sustainable alternative to conventional polymer foams without compromising vibration durability.

## 5. Conclusions

This study comprehensively investigated the vibration-induced degradation mechanisms of corrugated cardboard used as stabilizing packaging material for home appliances during transportation. The experimental approach enabled dynamic vibration durability experiments to elucidate how random vibration inputs affect the mechanical support characteristics over time. In the absence of vibration, stress relaxation occurred following a predictable exponential decay, converging to residual stress levels sufficient to maintain support. However, this behavior alone did not account for the progressive damage mechanisms observed in real transport vibration exposure scenarios. When subjected to sustained random vibration excitation, the packaging materials exhibited a clear exponential reduction in effective support stiffness. This reduction accelerated with increasing vibration magnitude and exposure duration. Notably, the stiffness degradation was strongly correlated with the material loss factor, indicating that viscoelastic and frictional damping mechanisms contributed to the observed fatigue behavior. The experimental results demonstrated that the low-frequency deformation modes of the refrigerator structure were the primary drivers of cumulative damage and eventual detachment of internal shelves. Vibration exposure caused the permanent deformation and wear of the stabilizer package, decreasing its effective length and reducing stiffness over time. This degradation trend was reliably captured by monitoring the frequency response of a cantilever beam supported by the test specimens, providing a quantitative indicator of structural integrity loss. Furthermore, statistical analysis of the random load cycles using auto-spectral density methods and Basquin’s power law allowed the prediction of fatigue life and estimation of failure probability under various excitation conditions. The study found that cardboard and EPS support exhibited similar degradation rates, suggesting that corrugated cardboard can serve as a viable, environmentally sustainable alternative to conventional polymer formats without significant compromise in vibration durability. Overall, this work highlights the importance of dynamic testing to evaluate packaging performance under realistic transport conditions. The vibration durability test methodology established in this study provides a practical framework for assessing packaging reliability and optimizing material selection.

## Data Availability

The original contributions presented in this study are included in the article material. Further inquiries can be directed to the corresponding author.
